# Multimodal Management of Extreme Hypertriglyceridemia in a Child with Recurrent Pancreatitis: Clinical Challenges and Solutions

**DOI:** 10.3390/jcm15020636

**Published:** 2026-01-13

**Authors:** Elena-Lia Spoială, Carmen Oltean, Ioana Vasiliu, Gabriela Paduraru, Diana-Claudia Danilă, Gabriela Ghiga, Maria Țugui, Lacramioara Ionela Butnariu, Elena Cojocaru, Laura Mihaela Trandafir

**Affiliations:** 1 Grigore T. Popa University of Medicine and Pharmacy, 700115 Iasi, Romania; elena-lia.spoiala@umfiasi.ro (E.-L.S.); ioana.vasiliu@umfiasi.ro (I.V.); lupu.diana-claudia@email.umfiasi.ro (D.-C.D.); gabriela.ghiga@umfiasi.ro (G.G.); ionela.butnariu@umfiasi.ro (L.I.B.); elena2.cojocaru@umfiasi.ro (E.C.); laura.trandafir@umfiasi.ro (L.M.T.); 2 Sfânta Maria Emergency Hospital for Children, 700309 Iasi, Romania; oltean_carmen@yahoo.com (C.O.); maria.tugui@email.umfiasi.ro (M.Ț.)

**Keywords:** hypertriglyceridemia, medium-chain triglycerides, children, xanthogranulomatous pancreatitis

## Abstract

**Background:** Severe hypertriglyceridemia (SHTG) in children is a rare but clinically significant disorder associated with recurrent acute pancreatitis and substantial morbidity. Early identification and prompt management are essential to prevent pancreatic and systemic complications. **Methods:** We report the case of an 11-year-old female with a history of xanthogranulomatous pancreatitis who presented with extreme hypertriglyceridemia, with fasting triglyceride levels exceeding 4000 mg/dL. **Results:** The patient was treated acutely with continuous intravenous aspart insulin (0.1 U/kg/hour) and adjusted 10% glucose infusion, with hourly glucose and potassium monitoring, leading to a rapid and marked reduction in triglyceride levels—55% reduction within the first 24 h, 76% at 48 h, and 82% after 96 h of treatment. No hypoglycemia or other adverse effects were observed. Nutritional management included a low–long-chain triglyceride (LCT) diet enriched with medium-chain triglycerides (MCTs) and omega-3 fatty acids, providing essential calories while minimizing chylomicron production. Over a 12-month follow-up, the patient remained asymptomatic, with sustained lipid normalization and no recurrence of pancreatitis. **Conclusions:** This case underscores the therapeutic value of combining pharmacologic and dietary strategies in pediatric SHTG. Evidence from pediatric and adult studies supports the role of insulin infusion for acute triglyceride lowering and MCT-based nutritional therapy for long-term control. Our findings highlight the need for early, individualized, and multidisciplinary management and emphasize the potential future role of emerging targeted therapies in addressing refractory pediatric hypertriglyceridemia.

## 1. Introduction

Severe hypertriglyceridemia (SHTG), commonly defined as fasting triglyceride levels ≥ 500 mg/dL—particularly when exceeding 1000 mg/dL—is a recognized risk factor for both acute and recurrent pancreatitis in children, although it remains distinctly rare in this age group [1]. Most pediatric cases of dyslipidemia are secondary to risk factors such as obesity, insulin resistance, or medication use; however, markedly elevated triglyceride levels (typically >1000 mg/dL) are often indicative of primary or genetic lipid disorders, including familial chylomicronemia syndrome [2] and multifactorial chylomicronemia syndrome [3]. Primary genetic lipid disorders, including familial chylomicronemia syndrome (FCS), represent rare causes of severe hypertriglyceridemia and are typically associated with inherited defects in pathways responsible for triglyceride metabolism, most often involving lipoprotein lipase-related mechanisms. These conditions are characterized by persistent and marked triglyceride elevations from an early age. In contrast, multifactorial chylomicronemia syndrome (MCS) is considerably more frequent and arises from the interplay between polygenic susceptibility and secondary metabolic or environmental factors. In clinical practice, differentiation between these entities relies mainly on phenotypic characteristics, age at onset, biochemical severity, and the exclusion of secondary causes, while genetic investigations may be used selectively to support the diagnosis when clinically and biologically indicated [4].

From a clinical perspective, mild to moderate hypertriglyceridemia (HTG), typically driven by elevated very low-density lipoprotein (VLDL), is linked to an increased risk of early-onset cardiovascular disease. In contrast, severe HTG, characterized by the accumulation of chylomicrons, significantly heightens the risk of acute pancreatitis [5]. Excessive chylomicrons markedly increase plasma viscosity and reduce pancreatic microcirculatory flow, leading to capillary stasis, local ischemia and acinar injury in the pancreas [6]. Also, chylomicrons and other triglyceride-rich lipoproteins, such as VLDLs that enter the pancreatic microcirculation, are acted upon by pancreatic lipase, an enzyme that hydrolyzes triglycerides into free fatty acids (FFAs). When TG levels are extremely high, the amount of FFAs released can exceed the binding capacity of albumin, resulting in high concentrations of unbound FFAs. These unbound FFAs have detergent-like properties that disrupt cell membranes, injure vascular endothelium, activate inflammatory pathways, and induce acinar cell damage, all of which contribute to the development and severity of pancreatitis [7]. Beyond direct cytotoxicity, elevated FFAs may impair mitochondrial function, disturb intracellular calcium homeostasis, and stimulate pro-inflammatory signaling in pancreatic acinar cells, further propagating tissue injury [8]. Moreover, the risk of pancreatitis is considered to be 5% when serum triglyceride levels (TG) are above 1000 mg/dL (11.3 mmol/L) and can climb up to 10–20% when levels exceed 2000 mg/dL (22.6 mmol/L) [9]. Furthermore, elevated TGs are frequently associated with nonalcoholic fatty liver disease, especially in obese or insulin-resistant children [10].

Timely recognition and intervention are essential, given the increased morbidity and potential mortality associated with pancreatitis in youth [11]. Management of SHTG in children requires a multifaceted approach. In the acute setting, the primary goal is rapid triglyceride reduction to mitigate pancreatic inflammation and prevent further episodes [12]. Continuous intravenous insulin infusion has been shown to be effective in facilitating triglyceride clearance by upregulating lipoprotein lipase activity, even in nondiabetic individuals [13]. Beyond the acute phase, long-term management focuses on dietary modification—most commonly fat restriction and the use of medium-chain triglyceride (MCT)-enriched formulas [14]—as well as supplementation with omega-3 fatty acids to sustain lipid control [15]. Implementing and maintaining these interventions can be especially challenging in the pediatric population, given age-related nutritional needs, adherence difficulties, and limited therapeutic options [16].

Herein, we report the case of an 11-year-old female presenting with extreme hypertriglyceridemia, which resulted in recurrent pancreatitis with fasting triglyceride levels peaking above 4000 mg/dL. Acute management included continuous intravenous insulin infusion and nutritional therapy incorporating an MCT-enriched milk formula. Long-term care comprised a specialized low-LCT (long-chain triglyceride) dietary regimen supplemented with omega-3 fatty acids. Over a 12-month follow-up, the patient remained asymptomatic, with progressive lipid profile normalization and no recurrent pancreatitis—illustrating the benefit of a multimodal therapeutic approach. By integrating a rare pancreatic pathology with severe pediatric dyslipidemia, this case underscores the importance of combining pharmacologic and nutritional interventions in the effective management of SHTG in children.

## 2. Case Report

We present the case of an 11-year-old female with a history of xanthogranulomatous pancreatitis and dyslipidemia, who was admitted to our Gastroenterology Department for clinical and laboratory reevaluation. At the time of the initial presentation, at the age of 10 years, the patient was assessed for acute-onset diffuse abdominal pain and loss of appetite. Abdominal ultrasound showed a liver of normal size with homogeneous texture, without focal lesions. The pancreas showed no signs of acute necrosis or fluid collections, and no ascites or biliary tract abnormalities were detected. Abdominal contrast-enhanced CT demonstrated a pancreatic mass localized to the distal pancreas, which prompted surgical intervention. Initial laboratory tests revealed elevated serum amylase and lipase, whereas alpha-fetoprotein, carcinoembryonic antigen (CEA), and carbohydrate antigen 19-9 (CA19-9) were within normal range. Histopathological examination following distal pancreatectomy revealed foamy histiocytes with lymphoplasmacytic infiltration, findings highly suggestive of xanthogranulomatous pancreatitis. Postoperatively, the patient experienced two episodes of acute pancreatitis, both clearly linked to marked hypertriglyceridemia. During these subsequent post-surgical episodes, abdominal CT revealed pancreatic inflammation without evidence of pancreatic necrosis, pseudocyst formation, or organized peripancreatic fluid collections. No biliary abnormalities were identified.

At the current admission, the patient was underweight, with a weight of 29 kg (−2.56 SD), height of 148 cm (−0.94 SD), and a BMI of 13.2 kg/m^2^ (1st percentile). No other pathological signs were present on clinical examination. Initial laboratory findings revealed mild eosinophilia and mild normocytic normochromic anemia. Liver and kidney function tests, as well as serum and urinary amylase and serum lipase levels, were within normal limits. Thyroid function, assessed by TSH and FT4, was also within normal limits. However, significant dyslipidaemia was identified under fasting conditions: total cholesterol: 316 mg/dL (reference: 119–212 mg/dL), HDL-cholesterol: severely reduced at 15 mg/dL (reference: >40 mg/dL), LDL-cholesterol: within normal limits, VLDL-cholesterol: markedly elevated at 581.2 mg/dL (reference: <32 mg/dL), serum triglycerides: 2906 mg/dL (reference: 44.2–256.53 mg/dL), peaking at 4034 mg/dL during the hospitalization. Lipoprotein electrophoresis was subsequently performed (after the initiation of specific treatment, as mentioned below) and showed no pathological findings (Table 1). Apolipoprotein B (apoB) levels were within the normal range: 0.9 g/L (reference range: 0.63–1.14 g/L).

Secondary causes of hypertriglyceridemia were systematically assessed and excluded as follows:

- Lifestyle and nutritional factors: Excessive intake of high-fat or high-sugar diets and sedentary behavior were unlikely contributors, as the patient was underweight (BMI 13.2 kg/m^2^) and reported a balanced diet.
- Endocrine and metabolic disorders:
  - Diabetes mellitus: Fasting glucose and HbA1c were within normal limits.
  - Hypothyroidism: TSH and free FT4 were normal.
  - Cushing’s syndrome/steroid excess: No clinical signs of hypercortisolism; the patient was not on corticosteroids.
  - Polycystic ovary syndrome (PCOS)/other hormonal disorders: Not applicable given age and normal pubertal development.
  - Metabolic syndrome/obesity-related insulin resistance: The patient was underweight, making obesity-related causes highly unlikely.

- Renal and hepatic conditions:
  - Nephrotic syndrome/chronic kidney disease: Urinary protein excretion and renal function tests (urea, creatinine) were normal.
  - Liver disease: Liver function tests (ALT, AST, ALP, bilirubin, GGT) were within reference ranges; abdominal ultrasound showed no structural liver disease.

- Autoimmune, systemic, or hematologic disorders: Complete blood count, ESR, CRP, complement levels (C3, C4), anti-nuclear antibodies (ANA), and anti-double-stranded DNA antibodies (anti-ds DNA).
- Neoplasia: Serum tumor markers (alpha-fetoprotein, CEA, and CA19-9) within normal range. In addition, histopathological examination of the pancreatic biopsy revealed no atypical or malignant cells.
- Medications/exogenous substances: The patient was not taking any drugs known to increase triglycerides, including corticosteroids, estrogens, beta-blockers, thiazides, atypical antipsychotics, retinoids, immunosuppressants, or parenteral nutrition.
- Alcohol consumption: Not applicable for age.
- Other clinical contexts:
  - Pregnancy: Not applicable.
  - Acute or chronic severe illness/sepsis: No evidence of infection or other acute illness at the time of assessment.

In this context, given the complete lipid panel, including lipoprotein electrophoresis and apolipoprotein B measurement—both within normal limits—a diagnosis of severe primary hypertriglyceridemia associated with recurrent acute pancreatitis was considered. Due to the diagnosis of mixed dyslipidaemia with markedly elevated triglyceride levels (Table 2), intravenous fast-acting insulin therapy (insulin aspart) was initiated at a rate of 0.1 U/kg/hour, equivalent to 3 U/hour. The insulin was prepared by diluting 50 units of insulin in 50 mL of 0.9% saline and administered at a rate of 3 mL/hour. This was accompanied by continuous 10% glucose infusion as oral feeding was temporarily suspended. The dextrose infusion was adjusted with concomitant titration of insulin to maintain optimal glycemic control. The target glucose range was maintained between 90 and 150 mg/dL, with no episodes of hypoglycemia (<70 mg/dL) during therapy. Throughout the treatment, hourly blood glucose and serum potassium levels were monitored to avoid iatrogenic hypoglycemia (defined as a glucose level less than 70 mg/dL) and hypokalemia (defined as serum potassium levels below 3.5 mEq/L. Insulin infusion was continued until serum triglycerides fell below 500 mg/dL, then gradually tapered over 24–48 h; subcutaneous insulin was not required, as glycemic control remained stable. No additional triglyceride-lowering agents, including heparin, fibrates, or omega-3 fatty acids, were administered during the acute phase.

**Figure 1:**
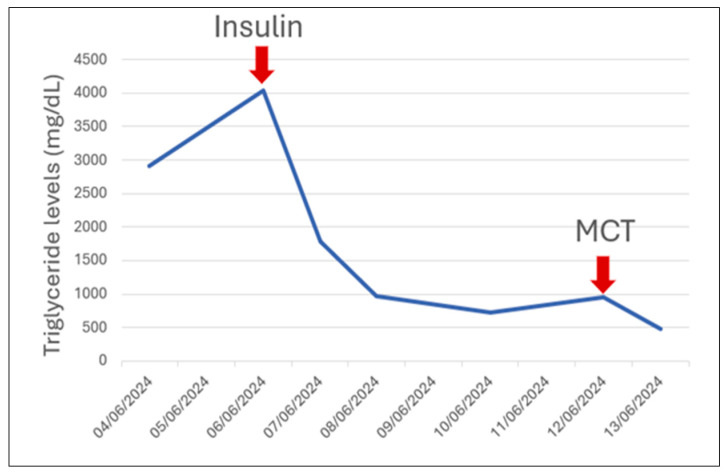
The dynamics of triglyceride levels in our patient.

Following the initiation of therapy, a significant reduction in serum triglyceride levels was observed, reaching 477 mg/dL. Insulin administration resulted in a 55% decrease in triglyceride levels within the first 24 h, a 76% reduction at 48 h, and an 82% decrease after 96 h of treatment, with no episodes of hypoglycemia or other adverse effects observed during treatment. (Figure 1). In addition to continuous insulin infusion—which is known to enhance lipoprotein lipase activity and thereby promote more rapid clearance of serum triglycerides—a powdered milk formula enriched with medium-chain triglycerides (MCTs) was introduced. This multimodal therapeutic strategy led to a further, clinically meaningful reduction in serum triglyceride concentrations.

Upon hospital discharge, the patient was transitioned to home-based nutritional therapy using a powdered milk formula enriched with MCTs, linoleic acid, α-linolenic acid, docosahexaenoic acid (DHA), and arachidonic acid (ARA). The formula was low in total fat and specifically reduced in long-chain triglycerides (LCTs). This dietary regimen was further supported by increased omega-3 fatty acid intake.

**Figure 2:**
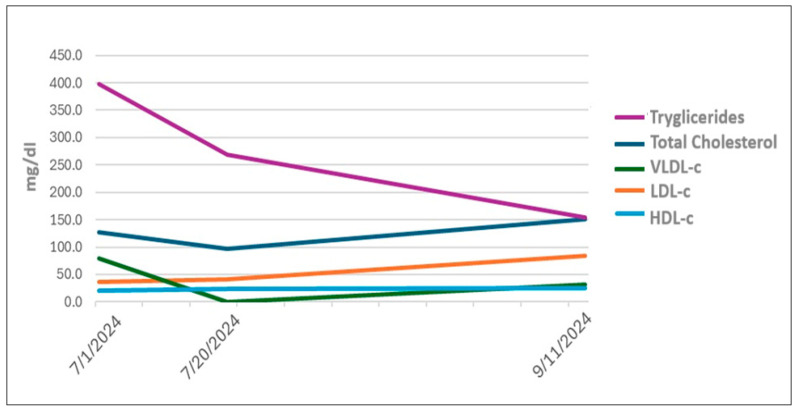
Progressive normalization of the lipid profile achieved via a combined management strategy.

At the 12-month follow-up, the patient remained asymptomatic and in excellent general condition, with no recurrence of pancreatitis or significant lipid abnormalities. The lipid profile demonstrated progressive normalization (Figure 2), highlighting the effectiveness of the combined dietary and pharmacologic approach in the management of severe hypertriglyceridemia. Follow-up imaging during clinical remission did not demonstrate features of chronic pancreatitis or pancreatic structural complications.

**Figure 3:**
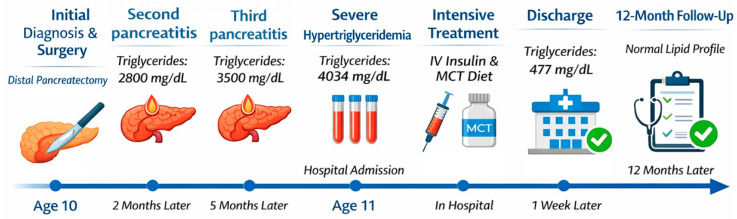
Schematic timeline of the patient’s clinical course.

Figure 3 summarizes the clinical course of the patient, illustrating key events including distal pancreatectomy, episodes of post-surgical acute pancreatitis, intensive treatment with IV insulin and MCT-enriched nutrition, discharge, and 12-month follow-up (Figure 3).

We acknowledge that genetic evaluation for familial chylomicronemia syndrome or other monogenic hypertriglyceridemias was not performed due to limited availability, high associated costs, and parental reluctance, particularly as the patient demonstrated a favorable clinical course under multimodal management. Also, the patient’s clinical presentation and laboratory profile (markedly elevated triglycerides with normal apoB, absence of chylomicronemia on lipoprotein electrophoresis, and lack of secondary lipid abnormalities) strongly suggested primary severe hypertriglyceridemia rather than a syndromic or secondary disorder. It was mutually agreed with the family that genetic testing would be considered in the event of recurrence of pancreatitis or severe hypertriglyceridemia during subsequent follow-up.

## 3. Discussion

Severe hypertriglyceridemia (HTG) is an uncommon but well-recognized cause of acute pancreatitis (AP) in children [1]. Most pediatric cases with HTG-AP involve TG levels >1000 mg/dL and often have genetic or secondary predisposing factors [1]. Several case reports illustrate this: For example, Bălănescu et al. (2013) described an 11-year-old girl with AP and TG ~1200 mg/dL [18] and Lilley et al. (2017) reported a 7-year-old with TG 2191 mg/dL and recurrent pancreatitis due to autoimmune LPL deficiency [19]. Lawson et al. documented a child with leukemia who developed pegaspargase-induced SHTG (TG 4640 mg/dL), which was successfully lowered with insulin [20]. These cases underscore that pediatric SHTG (often familial or drug-induced) can reach very high levels and precipitate pancreatitis. Moreover, published pediatric cases of hypertriglyceridemia-induced acute pancreatitis illustrate substantial heterogeneity with respect to age at presentation, etiology, therapeutic approach, and clinical outcomes (Table 3). Reported cases span a wide pediatric age range, from neonates and young infants with primary genetic disorders to adolescents with secondary hypertriglyceridemia related to insulin deficiency in type 1 diabetes mellitus. Peak triglyceride levels varied markedly, from approximately 1000 mg/dL in primary forms to extreme elevations exceeding 17,000 mg/dL in secondary cases associated with diabetic decompensation. Acute management strategies differed according to disease severity and underlying etiology. Supportive care alone was sufficient in selected neonatal cases with moderate triglyceride elevations, whereas older children and adolescents with severe hypertriglyceridemia more frequently required continuous intravenous insulin infusion, sometimes combined with fibrates or plasmapheresis to achieve rapid triglyceride reduction. Notably, insulin therapy was effective across both primary and secondary hypertriglyceridemia, supporting its role as a first-line acute intervention when urgent triglyceride lowering is required. In our patient, continuous intravenous insulin infusion combined with a medium-chain triglyceride (MCT)-enriched, low long-chain fat diet resulted in a rapid and sustained reduction in TG levels, without episodes of hypoglycemia or electrolyte disturbances, and prevented multisystem complications, highlighting the effectiveness of this approach in severe pediatric hypertriglyceridemia.

Acute management of HTG-AP generally follows standard pancreatitis care (aggressive IV fluids, bowel rest, analgesia) plus specific TG-lowering interventions [1]. Most therapeutic strategies for pediatric severe hypertriglyceridemia are informed by small observational case series or case reports, and extrapolation from adult studies, as randomized controlled pediatric trials are largely lacking.

**Figure 4:**
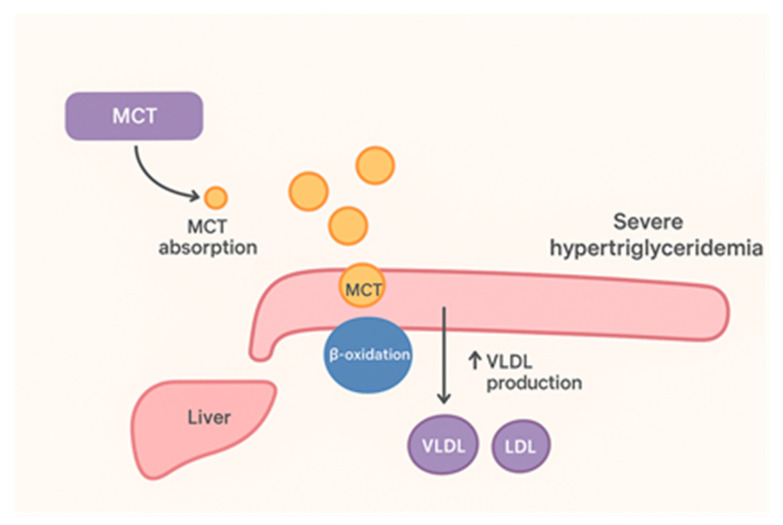
Role of MCTs in severe hypertriglyceridemia. (This schematic illustrates the distinct metabolic pathways of medium-chain triglycerides (MCTs) compared to long-chain triglycerides (LCTs) in the context of severe hypertriglyceridemia. Unlike LCTs, which require bile salts and pancreatic lipase for digestion and are packaged into chylomicrons that enter the lymphatic system and increase plasma triglycerides, MCTs (typically C8–C10 fatty acids) bypass this process. They are directly absorbed into the portal circulation, transported to the liver bound to albumin, and undergo rapid β-oxidation to provide energy or ketone bodies. As a result, MCTs supply calories without substantially contributing to chylomicron formation or exacerbating hypertriglyceridemia, making them a critical dietary component in the nutritional management of pediatric patients with severe hypertriglyceridemia.) Created by E.-L. Spoială et al., via BioRender, https://app.biorender.com/, on 10 November 2025.

Besides pharmacological interventions, nutritional therapy is critical: enteral or parenteral fat should be minimized, and MCT (medium-chain triglyceride)-based formulas are used to provide calories without chylomicron production [26]. The use of these MCT-based formulas in children is supported primarily by physiologic rationale, long-standing clinical experience, and small pediatric case series rather than randomized controlled trials. In our patient, as in prior reports, an MCT-enriched, low-long-chain fat diet was employed. First-line interventions emphasize strict dietary fat restriction to prevent excessive chylomicronemia and lower pancreatitis risk [27]. Guidelines for genetic chylomicronemia (familial chylomicronemia syndrome, FCS) recommend very-low-fat diets (often ≤15% of energy) and multiple daily meals to keep fasting triglycerides (TG) below 1000 mg/dL, above which chylomicrons and pancreatitis risk markedly increase [28]. In practice, achieving such TG targets in young children requires careful balance: fat must be limited while providing adequate calories and essential fatty acids for growth. Thus, nutritional therapy often pairs fat restriction with specialized supplements (e.g., omega-3 fatty acids) and, where available, medium-chain triglyceride (MCT) oil [29]. MCTs provide calories without increasing chylomicron production, forming the basis of nutritional therapy in pediatric SHTG [30]. To better illustrate the nutritional rationale, Figure 4 outlines the distinct metabolic handling of medium-chain triglycerides (MCTs) compared with long-chain triglycerides (LCTs), highlighting how MCTs bypass chylomicron formation and thereby provide an important caloric source without exacerbating hypertriglyceridemia in affected children.

Notably, commercial “MCT oil” (prescription medium-chain triglyceride oil) is required, since natural oils like coconut oil contain lauric acid (C12) that behaves partly like LCT and can still elevate chylomicrons [27]. Pediatric case series support the utility of MCT-based diets in genetic hypertriglyceridemia, though outcomes vary. Gracey et al. (1970) reported that “hyperchylomicronaemia responds well to dietary treatment with MCT” [31], reflecting early recognition of MCT benefits. More recent cohorts of children with monogenic chylomicronemia (e.g., ApoC-II deficiency) confirm this strategy: Yoldas Celik et al. described 12 children managed with a very-low-fat diet supplemented by MCT oil and omega-3 fatty acids [29]. In that series, MCT and fat restriction were used universally, yet over half of the patients still experienced pancreatitis episodes [29]. Similarly, a recent infant with FCS was treated from early on with an MCT-based formula and a fat-free diet; her TG initially fell, but she still developed pancreatitis at 2 months old until gemfibrozil was added [22]. These pediatric reports illustrate that while MCT diets form the backbone of care, many patients eventually require adjunctive therapy to maintain TG control. Fish oil supplements (EPA/DHA) are commonly added as adjunctive therapy. In adults, omega-3s lower TG by reducing hepatic VLDL synthesis; similar effects are presumed in children, though pediatric data are limited [27]. Since fat is restricted, close monitoring of essential fatty acid status and fat-soluble vitamins is essential [32].

Although diet is primary, fibrates or other TG-lowering drugs may be considered in children with very high TG (usually >500 mg/dL) or pancreatitis risk [33]. For example, gemfibrozil has been used in infants with FCS when diet alone was insufficient [22]. Evidence for omega-3 fatty acids in children is limited to small studies and case series, whereas triglyceride-lowering efficacy is well established in adult randomized trials; pediatric use therefore remains largely extrapolated from adult data. Table 4 provides an insight into the classical therapies proposed for hypertriglyceridemia.

**Figure 5:**
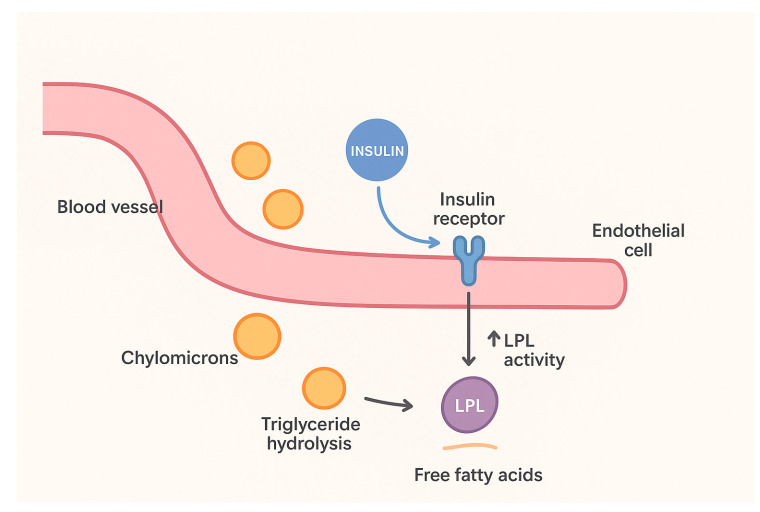
Insulin-mediated triglyceride clearance (This figure depicts the mechanism by which insulin lowers plasma triglycerides in severe hypertriglyceridemia. Circulating chylomicrons and very-low-density lipoproteins (VLDL) interact with endothelial cells expressing lipoprotein lipase (LPL). Insulin binds to its receptor and upregulates LPL expression and activity, thereby promoting hydrolysis of triglycerides into free fatty acids. These fatty acids are subsequently taken up by adipose and muscle tissue for storage or energy utilization. The net effect is enhanced clearance of triglyceride-rich lipoproteins from plasma, leading to a rapid reduction in circulating triglyceride levels and decreased risk of pancreatitis.) Created by E.-L. Spoială et al., via BioRender, https://app.biorender.com/, on 10 November 2025.

Insulin infusions or plasmapheresis are reserved for acute severe hyperTG. In our case, we used intravenous insulin for rapid TG reduction. In this context, interpretation of lipid phenotyping results requires careful consideration of the timing of sampling in relation to acute triglyceride-lowering therapy. In our patient, early initiation of continuous intravenous insulin infusion resulted in a rapid and sustained reduction in serum triglyceride levels, without metabolic complications. Under these conditions, chylomicron fractions may appear within the normal range despite previously extreme triglyceride levels, thereby masking prior chylomicronemia. Moreover, insulin upregulates endothelial lipoprotein lipase (LPL), accelerating the hydrolysis of triglyceride-rich lipoproteins (chylomicrons and VLDL) and thereby lowering serum TG [36]. Grisham et al. noted that insulin infusion can enhance LPL activity and clear chylomicrons, and that IV administration allows fine glucose control [1]. Dosing recommendations (e.g., 0.1–0.3 U/kg/h with dextrose to maintain euglycemia) have been proposed to maximize TG clearance while avoiding hypoglycemia [37]. In practice, insulin produces dramatic TG reductions; for instance, Lawson et al. reported an adolescent whose TG fell from 4640 to 522 mg/dL after 9 days of continuous insulin infusion [20]. In the review by Grisham et al., insulin infusions in HTG-AP cases achieved ~40–75% TG reduction within 2–3 days [1]. A small pediatric series similarly found that insulin-treated patients had ~40% TG drop at 24 h versus ~17% without insulin [38]. Clinically, insulin is indicated for SHTG both in diabetic and non-diabetic patients when urgent TG lowering is needed. By contrast, heparin (which transiently releases LPL) is generally not used long-term due to rebound TG rise and bleeding risk [39]. Figure 5 illustrates how insulin enhances lipoprotein lipase (LPL) activity, accelerating triglyceride clearance from chylomicrons and VLDL, thereby reducing plasma triglyceride levels.

When available, plasmapheresis is another intervention for severe HTG. Plasmapheresis can acutely remove 40–70% of plasma TG, but pediatric experience is limited [40]. Indications for apheresis include refractory SHTG with organ failure or multi-system inflammation [41]. However, apheresis is resource-intensive and not universally available; meta-analyses show rapid TG clearance but mixed effects on outcomes [42]. Importantly, if apheresis is not feasible, experts recommend aggressive medical therapy—notably continuous insulin infusion even in normoglycemic children. Insulin infusion is a well-documented, effective acute therapy in pediatric SHTG-AP (especially when delivered on a “two-bag” insulin/dextrose protocol) [43]. While plasmapheresis can produce rapid reductions in serum triglyceride levels, its use in pediatric patients is generally reserved for refractory severe hypertriglyceridemia. Evidence from a recent systematic review and meta-analysis by Piplani et al. (2024) demonstrated that insulin ± heparin therapy and plasmapheresis are equally effective in lowering triglycerides in patients with hypertriglyceridemia-induced acute pancreatitis [44]. Considering cost-effectiveness, convenience, and resource availability, insulin ± heparin therapy is often preferred in clinical practice, particularly in resource-limited settings. In our patient, a rapid and sustained reduction in triglycerides was achieved with continuous intravenous insulin infusion combined with MCT-enriched nutrition, without clinical evidence of organ failure or multisystem complications. Given the favorable response and the resource-intensive nature of plasmapheresis, it was judged unnecessary in this case and was not pursued.

Our multimodal approach—with insulin and MCT-based nutrition—parallels strategies reported in previous pediatric SHTG-AP cases [18,19,20]. However, the combination with xanthogranulomatous pancreatic lesions has not been documented, highlighting both the rarity and clinical relevance of our report. Long-term nutritional therapy remains essential for maintaining triglycerides below pancreatitis-risk thresholds while ensuring adequate caloric intake and essential fatty acids. The favorable outcome at 12-month follow-up emphasizes the effectiveness of early, individualized, evidence-based interventions.

While established therapeutic strategies such as insulin therapy and dietary modification remain the cornerstone of acute and long-term management, ongoing advances in lipid metabolism have prompted the development of novel targeted therapies. A variety of novel and investigational agents targeting different aspects of triglyceride metabolism are currently under evaluation (Table 5). These therapies include inhibitors of apolipoprotein C-III (APO-CIII) and angiopoietin-like protein 3 (ANGPTL3), fibroblast growth factor 21 (FGF21) analogs, peroxisome proliferator-activated receptor alpha (PPARα) agonists, and microsomal triglyceride transfer protein (MTP) inhibitors. Each drug class employs a distinct mechanism of action—ranging from antisense oligonucleotides (ASO) and small interfering RNA (siRNA) to monoclonal antibodies (MoAb) and receptor agonists—aimed at reducing triglyceride levels and associated complications. While some of these therapies demonstrate impressive efficacy, safety concerns, adverse effects, and limited data in pediatric populations remain important considerations. Table 5 summarizes key agents, their mechanisms, advantages, limitations, and current clinical status [45,46,47,48].

Beyond its association with acute pancreatitis, hypertriglyceridemia (HTG) is increasingly recognized as a contributor to multiple systemic complications. Chronically elevated triglyceride levels are implicated in the development of atherogenic dyslipidemia, characterized by elevated concentrations of small dense low-density lipoprotein (LDL) particles, reduced high-density lipoprotein (HDL), and elevated apolipoprotein B-100—all of which are established risk factors for premature cardiovascular disease [49]. Moreover, HTG frequently coexists with insulin resistance and type 2 diabetes mellitus, conditions that further exacerbate triglyceride overproduction and impair clearance, creating a vicious metabolic cycle [50]. These interrelated complications not only increase long-term morbidity but also complicate management, highlighting the importance of early recognition, comprehensive risk assessment, and tailored therapeutic strategies in patients with severe HTG. To our knowledge, no pediatric cases of xanthogranulomatous pancreatitis associated with severe hypertriglyceridemia have been reported so far. This lack of previously documented cases highlights the rarity and distinctiveness of our patient’s presentation. While xanthogranulomatous pancreatic lesions have been described only rarely and mostly in adults, hypertriglyceridemia-induced pancreatitis is a recognized entity in children.

## 4. Conclusions

Severe hypertriglyceridemia in children represents a rare but serious condition, most often revealed through episodes of recurrent pancreatitis. Early recognition and rapid triglyceride-lowering interventions are essential to reduce acute morbidity and prevent complications such as pancreatic necrosis or systemic inflammation. In the acute setting, continuous insulin infusion combined with strict dietary measures can achieve rapid biochemical improvement, as illustrated in our case. This highlights the importance of timely, evidence-based interventions even in pediatric patients without diabetes.

Long-term management, however, presents greater challenges. Nutritional therapy remains the cornerstone of treatment, with low long-chain triglyceride diets supplemented by medium-chain triglycerides and omega-3 fatty acids forming the foundation of care. Such approaches can reduce chylomicronemia while still providing sufficient caloric intake for growth and development. Nevertheless, adherence is difficult in the pediatric population, and dietary strategies alone are often insufficient to maintain triglycerides below the thresholds associated with pancreatitis risk. This underscores the need for continuous monitoring, family education, and multidisciplinary support.

Looking ahead, emerging pharmacologic therapies targeting APO-CIII, ANGPTL3, and other pathways hold promise for patients with refractory or genetically determined hypertriglyceridemia. While their efficacy in adults is increasingly well documented, pediatric safety and effectiveness data remain scarce. Until such evidence becomes available, management in children must rely on a multimodal approach that combines rigorous dietary interventions with carefully selected pharmacologic agents when necessary. Collaborative research and pediatric-specific clinical trials are crucial to expand the therapeutic arsenal and improve long-term outcomes in this vulnerable population.

## Figures and Tables

**Table 1:** Lipoprotein Electrophoresis.

Lipoprotein Fraction	Result (%)	Reference Range (%)
Alpha Lipoproteins	37.51	14–46
Pre-Beta Lipoproteins	27.8	6–40
Beta Lipoproteins	33.97	28–61.7
Chylomicrons	0.71	0–2

**Table 2:** Hypertriglyceridemia classification based on the Endocrine Society recommendations [17].

Age	Normal	Borderline	High	Very High	Severe	Very Severe
0–9 years	<75 mg/dL	≥75–99 mg/dL	≥100–499 mg/dL	≥500–999 mg/dL	≥1000–1999 mg/dL	≥2000 mg/dL
10–19 years	<90 mg/dL	≥90–129 mg/dL	≥130–499 mg/dL	≥500–999 mg/dL	≥1000–1999 mg/dL	≥2000 mg/dL

**Table 3:** Representative Pediatric Cases of Hypertriglyceridemia-Induced Acute Pancreatitis: Clinical Features, Management, and Outcomes.

Article (Author, Year)	Age at First Presentation (Sex)	Peak TG	Cause	Pharmacological Treatment	Nutritional Treatment	Outcome	Age and Status at Last Follow-Up/Control
Yıldız et al., 2020 [21]	37 days old (M)	1091 mg/dL	Primary	Supportive care only (no insulin reported)	NPO 48 h → gradual refeeding	TG reduction (level between 250 and 450 mg/dL)	~2 months (at discharge), resolution of pancreatitis
Mustafa et al., 2024 [22]	2 months old (F)	3226 mg/dL	Primary	IV fluids + gemfibrozil 70 mg/kg/day	MCT-enriched diet	TG decreased (to 1010 mg/dL at 8 weeks, 708.6 mg/dL at 10 weeks, 354.3 mg/dL at 2 years); pancreatitis resolved; no recurrence reported	6 years and 6 months old, asymptomatic, on low-fat diet with MCT oil-based milk, with no history of other hospital admissions
Sharma et al., 2017 [23]	4 years old (F)	13,846 mg/dL	Secondary (type 1 diabetes mellitus—T1DM)	Continuous IV insulin, then subcutaneous insulin	NPO → low-fat diet	TG reduced to 5609 mg/dL by day 3; to 490 mg/dL by day 14	≈4 years old, TG reduced to 90 mg/dL at 28-day follow-up
1	4 years 9 months old (M)	1359 mg/dL	Primary	Intravenous rehydration, antibiotics, statins, fibrates, and pancreatin supplements	NPO → gradual refeeding	Pancreatic necrosis and pseudocyst; improvement after drainage	≈4 years 10 months old, with TG reduced to 214 mg/dL
Valenzuela-Vallejo et al., 2022 [24]	14 years 11 months old (F)	4260 mg/dL	Primary	Continuous IV insulin 0.08 U/kg/h, fibrates; multiple plasmapheresis sessions	Strict low-fat diet	Sustained TG reduction and no further episodes reported	≈15 years 8 months old, with TG reduced to 495 mg/dL
Muñoz et al., 2024 [25]	15 years old (F)	17,580 mg/dL	Secondary (T1DM)	IV insulin infusion (0.05 U/kg/h); gemfibrozil 900 mg/day	NPO → fat-restricted diet	TG reduced to 519 mg/dL by day 6	≈16 years old, with complete anatomical resolution of pancreatic involvement at 1 year of follow-up
Present case	10 years old (F)	4034 mg/dL	Primary	Continuous IV insulin 0.1 U/kg/h	NPO → gradual refeeding, MCT-based nutrition	Rapid TG reduction; no organ failure; no recurrence during follow-up	≈12 years old, with TG reduced to 154 mg/dL

**Table 4:** Classical therapies for managing hypertriglyceridemia [34,35,36].

Type of Medication	Examples	Indications	Mechanism of Action	Effects on Lipid Profile	Adverse Reactions	Indications/Comments
HMG CoA (3-Hydroxy-3Methyl-Glutaryl Coenzyme A) reductase inhibitors (statins)	Atorvastatin Fluvastatin Lovastatin Pravastatin Rosuvastatin Simvastatin	Maintenance/ Preventive	- Inhibits cholesterol synthesis in hepatic cells; - Decreases cholesterol pool; - Upregulation of LDL receptors	- Mainly lowers LDL–C; - Some decrease in TG; - Modest increase in HDL–C	- Elevated hepatic transaminases and creatine kinase; - Myopathy with risk of rhabdomyolysis; - Severe renal impairment (CrCl < 30 mL/min); - Headache, nausea, sleep disturbances	Statin therapy is generally not initiated before age 10, except in select cases with high-risk family history, conditions, or multiple risk factors. In HTG, statins are often combined with fibrates for synergistic lipid-lowering effects.
Fibric acid derivatives	Fenofibrate Gemfibrozil	Maintenance/ Preventive	PPAR-α agonists; upregulate LPL and downregulate apoC-III, enhancing VLDL and TG catabolism; they may also reduce hepatic VLDL synthesis.	- Marked TG reduction; - HDL-C increase; - Minimal effect on LDL-C	- GI disturbances: nausea, bloating, cramping, dyspepsia, constipation; - Myositis; - Anemia; - Rash; - Myalgia; - Potential nephrotoxicity in cyclosporine-treated patients; - Risk of cholesterol gallstones	Avoid CrCl < 30 mL/min; monitor renal and hepatic function regularly. The use of fibrates in youths should be undertaken only with the assistance of a lipid specialist.
Nicotinic acid (extended release)	Niacin, extended release	Maintenance/ Preventive	- Inhibits adipose tissue lipolysis, reducing FFA availability; - Decreases hepatic VLDL and LDL synthesis; - Slows HDL catabolism	- Lowers TG, LDL-C, and Lp(a); - Increases HDL-C	- Flushing (prostaglandin-mediated), pruritus, headache, dry skin, GI upset (nausea, vomiting, diarrhea), myositis, hepatotoxicity; - May raise fasting glucose and uric acid	Contraindicated in active liver disease or unexplained elevated LFTs The use of niacin in youths should be undertaken only with the assistance of a lipid specialist.
Omega-3 fish oil	Ethyl esters’ Icosapent	Maintenance/ Preventive	Inhibits hepatic fatty acid and triglyceride synthesis; -Enhances β-oxidation; reduces VLDL production	- Lowers TG, - Increases HDL-C and LDL particle size; - May increase LDL-C	- Mild GI effects (eructation, dyspepsia, diarrhea 7–15%); - May potentiate antiplatelet/anticoagulant therapy;	Experience with fish oil in children is limited to small case series with no safety concerns identified.
Insulin		Acute Severe HTG	Enhances lipoprotein lipase (LPL) activity, promoting chylomicron clearance and rapid TG reduction	Rapid decrease in serum triglycerides.		IV insulin (0.05–0.1 U/kg/h) preferred over subcutaneous route in severe cases. Administer IV dextrose concurrently to prevent hypoglycemia.
Heparin		Acute Severe HTG	Transiently increases circulating LPL by mobilizing endothelial stores; - Prolonged use may deplete LPL, reducing efficacy.	Temporary TG reduction		Not recommended as monotherapy due to short-lived effect and risk of LPL depletion.

**Table 5:** Emerging therapies in hypertriglyceridemia [45,46,47,48].

Type of Medication	Examples	Target	Mechanism of Action	Advantages	Disadvantages	Comments
APO-CIII inhibitors	Volanesorsen	Liver	ASO	Lowering of TGs by over 70%, decreased pancreatitis	Thrombocytopenia Injection site reaction	Safety and efficacy in <18 s not established.
	Olezarsen		ASO	Significant reductions in TG levels	Liver and kidney abnormalities	Treatment of adults with FCS
	Plozasiran		siRNA	Reduces TG levels	Favorable safety profile	FDA Accepts Plozasiran NDA for Adult FCS Patients.
ANGPTL3 inhibitors	Evinacumab	Muscle, adipocytes	MoAb	Reduces plasma TG and LDL-C in an LDL receptor independent manner	Nausea Severe allergic reactions	As an adjunct to diet and other LDL-C-lowering therapies for the treatment of adult, pediatric, and adolescent patients aged 5 years and older with HoFH.
	Vupanorsen		ASO	Reduction in cholesterol and plasma TGs	Increase in serum transaminases	Development stopped
	Zodasiran		siRNA	Reduces TG levels	Patients with pre-existing diabetes experienced a temporary rise in glycated hemoglobin at the highest dose	Development stopped
FGF21 analogs	Pegozafermin		growth factor analog	Reduces TG levels	No major adverse events reported	Currently under investigation for the treatment of adults with NASH and severe hypertriglyceridemia
PPARα agonist	Pemafibrate	Liver, muscle, adipocytes		High selectivity of the receptor	Renal adverse events and risk of venous thromboembolism	Not approved in the US or EU for general use.
MTP inhibitor	Lomitapide	Liver, enterocyte		Reduces LDL levels	Increase in serum transaminases	Indicated for adults with HoFH. The safety and efficacy of lomitapide in children < 18 years have not been established.

APO—apolipoprotein; ASO—anti-sense oligonucleotide; siRNA—small interfering RNA; ANGPTL—angiopoietin-like proteins; FCS—familial chylomicronemia syndrome; MoAb—monoclonal antibody; HoFH—homozygous familial hypercholesterolemia; FGF21—Fibroblast Growth Factor 21; PPARα—Peroxisome Proliferator-Activated Receptor alpha; MTP—Microsomal Triglyceride Transfer Protein.

## Data Availability

The original contributions presented in this study are included in the article. Further inquiries can be directed to the corresponding author.

## Abbreviations

The following abbreviations are used in this manuscript:

ALA	Alpha-Linolenic Acid
ANGPTL3	Angiopoietin-Like Protein 3
ApoB	Apolipoprotein B
APO-CIII/APOC3	Apolipoprotein C-III
ARA	Arachidonic Acid
ASO	Antisense Oligonucleotide
BMI	Body Mass Index
CrCl	Creatinine Clearance
CVD	Cardiovascular Disease
DHA	Docosahexaenoic Acid
EPA	Eicosapentaenoic Acid
FCS	Familial Chylomicronemia Syndrome
FDA	Food and Drug Administration
FFA	Free Fatty Acids
FGF21	Fibroblast Growth Factor 21
FT4	Free Thyroxine
GI	Gastrointestinal
HDL-C	High-Density Lipoprotein Cholesterol
HoFH	Homozygous Familial Hypercholesterolemia
HTG	Hypertriglyceridemia
IV	Intravenous
LCT	Long-Chain Triglyceride
LDL-C	Low-Density Lipoprotein Cholesterol
LFTs	Liver Function Tests
LPL	Lipoprotein Lipase
MCS	Multifactorial chylomicronemia syndrome
MCT	Medium-Chain Triglyceride
MoAb	Monoclonal Antibody
MTP	Microsomal Triglyceride Transfer Protein
NDA	New Drug Application
NASH	Non-Alcoholic Steatohepatitis
NPO	Nihil per os
PPARα	Peroxisome Proliferator-Activated Receptor Alpha
SD	Standard Deviation
SHTG	Severe Hypertriglyceridemia
siRNA	Small Interfering RNA
TSH	Thyroid-Stimulating Hormone
T1DM	Type 1 diabetes mellitus
VLDL	Very Low-Density Lipoprotein
